# A combined debridement, antibiotics, and implant retention (DAIR) procedure with flap coverage for acute soft tissue defects following total knee arthroplasty: a retrospective study

**DOI:** 10.5194/jbji-9-241-2024

**Published:** 2024-10-29

**Authors:** Laia Boadas-Gironès, Marta Sabater-Martos, Marc Ferrer-Banus, Àlex Soriano-Viladomiu, Juan Carlos Martínez-Pastor

**Affiliations:** 1 Orthopedic Surgery and Traumatology, Hospital Clínic de Barcelona, C/ Villarroel 170, 08036 Barcelona, Spain; 2 Infectious Diseases, Hospital Clínic de Barcelona, C/ Villarroel 170, 08036 Barcelona, Spain

## Abstract

Acute soft tissue defects, such as persistent drainage, wound dehiscence, or necrosis, following total knee arthroplasty (TKA) can lead to the devastating complication of deep infection. Typically, when a medium-sized defect is present, a gastrocnemius flap is widely employed for soft tissue reconstruction due to its low morbidity and favourable functional outcomes.

When facing this situation, we should consider associating the coverage treatment with a debridement, antibiotics, and implant retention (DAIR) surgery procedure, in order to treat a possible acute infection, even when the diagnosis of infection is not clear.

We performed a retrospective study to compare TKA outcomes in patients with DAIR and flap procedures in the same surgical act against those who had received an isolated flap procedure for soft tissue reconstruction after an acute surgical wound defect. Patients had been identified from a prospectively collated TKA database.

Between 2005 and 2021, 18 patients met our inclusion criteria, with a mean follow-up of approximately 8 years. A medial gastrocnemius flap procedure was performed for 15 patients (83 %).

We compared the rates of infection clearance between the two groups. No differences in comorbidities or risk factors were observed between both groups. In the combination treatment group, 66.6 % of patients healed after treatment compared to 33.3 % in the isolated flap group.

Although no significant statistical differences were found, the association of DAIR with the muscle flap procedure is highly recommended in the treatment of acute soft tissue defects after TKA. Further studies with larger sample sizes are necessary to extrapolate these findings to the general population.

## 1 Introduction

Acute soft tissue defects such as persistent drainage, wound dehiscence, wound leakage, or necrosis following total knee arthroplasty (TKA) can lead to the devastating complication of periprosthetic joint infection (PJI).

The incidence of wound complications after a TKA is 20 %, with 0.33 % of TKA patients needing surgical treatment. After a wound complication, the risk of requiring major intervention may increase 5-fold [Bibr bib1.bibx16].

The risk factor for developing a knee wound complication may hinge on various factors related to the patient's health, including conditions such as diabetes, smoking (which increases the risk of bleeding and infection), and obesity (associated with an increased risk of dehiscence and deep-vein thrombosis). Additionally, local factors such as previous scars, major vessel trauma, haematoma, previous local infection, skin tension during closure, or previous irradiated skin can contribute to the risk of developing complications in knee wounds.

The McPherson [Bibr bib1.bibx3], joint-specific BACH (JS-BACH) [Bibr bib1.bibx6], and TNM for prosthetic joint infections (PJI-TNM) [Bibr bib1.bibx1] classifications can be useful in describing and predicting the risk of failure in periprosthetic infections, as they include descriptions of the conditions of soft tissues and the host's systemic conditions in their criteria.

When the defect cannot be directly closed or involves bone or metalwork exposure, a coverage treatment is necessary, and a plastic surgery expertise is required. Additionally, in cases of skin tension during TKA closure, even in the absence of a skin defect, coverage treatment could improve healing. This treatment will improve the delivery of oxygen and systemic antibiotics and act as an immune modulator in infected joints. Attempting joint reconstruction is futile if soft tissues cannot adequately cover the prosthesis.

Typically, when a medium-sized defect is present (about 4–6 cm) in the patellar or infra-patellar region with prosthesis or bone exposure, a medial gastrocnemius flap is employed. Previously, the soft tissue reconstruction type has been determined based on the location and Laing classification [Bibr bib1.bibx12]. The Laing classification had categorized lesions according to the severity of wound dehiscence, the depth affected, and the presence of prosthesis exposure. Nowadays, this classification is considered outdated since all wound-healing problems (persistent drainage, necrosis or dehiscence) after TKA are considered deep wounds, as there is not enough tissue to determine the difference between superficial and deep wounds around the knee. The gastrocnemius flap is widely used because of its low morbidity and high functional outcomes. The reported knee prothesis implant survival rate after a gastrocnemius flap in the literature is approximately 90 % after acute wound defect treatment [Bibr bib1.bibx16].

On the other hand, an early prosthetic joint dehiscence or the presence of persistent drainage after 3 weeks may be a sign of an acute prothesis joint problem. Furthermore, an open surgical wound presents a potential entrance for microorganisms that could contact the prosthesis and develop a prosthetic joint infection (PJI). In front of an acute joint infection in a well-fixed prothesis, a debridement, antibiotics, and implant retention (DAIR) procedure should be recommended.

In the scenario of an acute wound-healing problem, evaluation is typically conducted by different specialties (orthopaedic surgeons, plastic surgeons, and/or infectious disease specialists). A multidisciplinary analysis with a consensus decision should be performed in such situations to consider the association of coverage treatment with a DAIR procedure. This dual approach aims to address potential acute PJI in patients with soft tissue defects, even when the infection diagnosis remains uncertain. We could not find any studies comparing the efficacy of this combined approach within a single surgical act versus isolated treatment for soft tissue defects.

Our hypothesis was that combining a DAIR procedure with soft tissue reconstruction surgery, such as a muscle flap, in the same surgical act could improve the likelihood of prosthesis survival in cases of acute wound defects (dehiscence or necrosis) following TKA, even when the infection has not been confirmed.

## 2 Material and methods

This retrospective study compares total knee arthroplasty (TKA) infection clearance in patients who underwent debridement, antibiotics, and implant retention (DAIR) with a concurrent flap procedure versus those who received an isolated flap procedure for soft tissue reconstruction following an acute surgical wound defect after TKA surgery.

Patients were identified from a prospectively collated TKA and flap database between 2005 and 2021. The inclusion criteria encompassed all patients diagnosed with acute wound dehiscence or acute wound complications after TKA, including those who underwent primary or revision prosthesis or arthrodesis, and received a musculocutaneous flap procedure for soft tissue reconstruction. Patients who received muscle flaps for knee extensor mechanism loss reconstruction or musculocutaneous flaps for prophylactic wound complication treatment in chronic TKA joint infection were excluded, with or without the presence of a sinus tract.

Healing or TKA infection clearance was defined as the presence of the original prosthesis after soft tissue reconstruction intervention within 2 years, without subsequent DAIR or suppressive antibiotic treatment.

Eighteen patients meeting the inclusion criteria were identified, with a median follow-up of approximately 8 years. A medial gastrocnemius flap was performed for 15 patients (83 %), a lateral gastrocnemius flap was performed for 2 patients (11 %), and a latissimus dorsal flap was performed for 1 patient (6 %).

Our sample was categorized into two groups: FLAP with DAIR (patients receiving a DAIR procedure with polyethylene exchange and flap coverage concurrently, i.e. proactive DAIR) and FLAP without DAIR (patients undergoing isolated soft tissue reconstruction treatment without a concurrent DAIR procedure).

Surgical decisions were made at the discretion of the surgeon or based on the clinical evolution of each patient.

The combination treatment group (FLAP with DAIR) included 12 patients, while the isolated group (FLAP without DAIR) included 6 patients. No differences in comorbidities or risk factors were observed between both groups, including sex, age, body mass index, ASA (American Society of Anesthesiologists classification), KLICC score (Tornero et al., 2015), smoking, fever, pain, redness, wound drainage, necrosis, knee defect, or type of knee prosthesis (primary or revision).

Secondary outcomes such as the type of acute wound defect, previous local risk factors, comorbidities, type of flap performed, type of knee prosthesis, and microbiological culture results obtained during various surgical procedures were reported.

The TKA infection clearance was compared for patients with the combination treatment versus those receiving an isolated flap procedure for soft tissue reconstruction after an acute surgical wound defect after TKA surgery. Additionally, the reasons for failure were described. Sub-analysis of reported data and final outcomes had been done, in order to describe the risk of failure.

In this study, we define the medical term “coverage” as the process of covering a defect or wound to protect underlying structures and promote healing. This is particularly necessary in cases where there is a loss of skin or soft tissue due to trauma, surgery, infection, or chronic wounds, involving procedures such as skin grafts, local flaps, or free flaps. We define “reconstruction” as the process of rebuilding or restoring the form and function of a body part that has been damaged or is missing. While these terms may overlap in some references, they have distinct meanings in other contexts. Therefore, we considered the differences between these terms in our narration.

Statistical analyses were performed using Jamovi version 2.3.19.0. Demographic data were compared between groups using Fisher's exact test or a t test for non-parametric variables. Continuous variables were expressed with median and interquartile range (IQR), and dichotomous variables were expressed with absolute numbers and percentages. Multinomial logistic regression analysis was performed for multivariate outcomes. Risk of failure was evaluated using relative risk and its 95 % confidence interval (CI), with statistical significance set at p<0.05 .

## 3 Results

The median age of our population was 72 years (range: 54–82), with a median follow-up of 7 years (interquartile range (IQR): 2–19).

Among the 18 patients, 8 were male. Four patients had previously been diagnosed with diabetes, and six were taking oral anticoagulants. Two patients had undergone a previous flap procedure. In 9 out of 18 patients, flap surgery was performed over a revision prosthesis; 83.3 % had persistent wound drainage, and 72 % had cutaneous necrosis (Table A1).

In the FLAP without DAIR group, five out of six patients had positive cultures during flap surgery. Two patients (33 %) had positive cultures for *Enterobacter* bacteria (*Escherichia coli* (E. Coli) and *Klebsiella*), two patients (33 %) had positive cultures for *Staphylococcus epidermidis*, and one patient had a positive culture for *Enterococcus*. One case showed persistence of the same microorganism, *Klebsiella pneumoniae*, from previous joint cultures.

In the FLAP with DAIR group, 3 out of 12 patients (25 %) had negative cultures during surgery, all of whom had negative cultures in previous arthrocentesis. Among those with positive cultures, *Enterococcus* was the most commonly identified microorganism type (Table B1).

The failure rate in our sample was 8 out of 18 patients (44.44 %). The main reason for failure was persistent infection after flap surgery, requiring a two-stage revision in 22 % of cases, i.e. two cases in each group. Three cases required amputation, two of which were in the FLAP without DAIR group.

Twelve patients were included in the FLAP with DAIR group: eight of them had infection clearance, two required a two-stage revision, one needed amputation, and one received a suppressive treatment to control the infection.

Six patients were included in the FLAP without DAIR group: two of them had infection clearance, two needed a two-stage revision, one required extremity amputation, and one remained on suppressive treatment.

In the combination treatment, 66.67 % of patients healed after treatment compared to 33.33 % in the isolated treatment group. Therefore, patients in the FLAP without DAIR group presented a higher failure rate with a relative risk of 2, although these differences were not statistically significant (95 % CI: 0.75–5.33; p=0.18 ) (Table C1).

We conducted a sub-analysis to describe the risk factors for failure. Although no statistical differences were observed between the type of flap and favourable outcomes, three out of four cases (75 %) that received a non-medial gastrocnemius flap failed (lateral gastrocnemius flap or latissimus dorsalis flap), all of whom had undergone previous flap surgeries (medial gastrocnemius flap).

The median number of previous knee surgeries, including primary TKA, before flap procedures was 2 (IQR: 1–7).

We observed a higher rate of failure in patients with multiple previous joint surgeries ( >2 surgeries), with a failure rate of 10 out of our sample of patients (55.56 %). The relationship between multiple previous surgeries and failure was statistically significant ( p<0.05 ) with a relative risk of 3.75 (95 % CI: 1.02–13.8). In 2 out of the 10 described cases (20 %), one of the procedures involved flap coverage.

We also analysed the impact of positive cultures during flap surgery on the final outcome (infection clearance). We found a positive relationship between negative cultures and favourable outcomes, although the differences were not statistically significant, with a relative risk of approximately 0.5 (95 % CI: 0.08–2.95).

## 4 Discussion

The successful condition of soft tissue over the knee is the main factor of success after a knee arthroplasty infection – hence the necessity for a correct treatment in acute wound defect (dehiscence or necrosis).

While skin blood supply is largely dependent on the terminal branches of the anterior anastomoses, there is a better blood supply originating medially. The poor vascularity of the skin over a total knee arthroplasty affects the risk of necrosis and subsequent healing rates postoperatively. In cases of poor healing of wounds or skin necrosis after TKA, early identification of the issue minimizes the risk of deeper infection.

Several operative techniques are described for addressing persistent open wounds, ranging from primary suturing after proper debridement of the lesion to fasciocutaneous flaps, pedicle rotational muscle flaps, and free flaps. The musculocutaneous flap creates a favourable environment with adequate tissue oxygen supply and effective immunologic and antibiotic delivery. The medial and/or lateral gastrocnemius flap is generally considered the first-line treatment of choice for knee defects following arthroplasty. The success of this approach is attributed to the main vascularity of the flap, with the medial sural artery providing a single pedicle around which the muscle can be rotated. Additionally, the coverage area can range from 30 to 50 cm, and the arc of rotation allows for reconstruction of, most commonly, anterior defects, thus addressing problems associated with the distal half of the incision without necessitating microsurgery techniques [Bibr bib1.bibx5].

[Bibr bib1.bibx16] advocate for favourable outcomes following the use of a medial gastrocnemius flap to address anterior soft tissue defects in knee arthroplasty infection treatment, reporting success rates around 52 %. However, their sample included patients with infected knee arthroplasties without distinguishing between acute or chronic infection, nor did they specify the debridement treatment received (DAIR, single exchange, or two-stage exchange).

In the study by [Bibr bib1.bibx18], only eight patients (30.8 %) achieved infection clearance after initial treatment and flap placement. [Bibr bib1.bibx15] observed that 40 % of their patients experienced persistent or recurrent infection during follow-up, with 32 % eventually requiring arthrodesis or above-the-knee amputation. In contrast, [Bibr bib1.bibx2] reported superior outcomes; among their series of 24 patients, only 13 % experienced persistent or recurrent infection after flap placement, with only one patient (4 %) undergoing amputation. Variability in results across studies may stem from differences in study populations and definitions of healing.

In our study, the sample was clearly defined as patients who underwent a flap procedure to address anterior knee acute defects after knee arthroplasty, with or without associated DAIR procedures, in the presence or absence of confirmed periprosthetic joint infection. We observed a success rate of approximately 55.6 %, defining healing as the presence of the original prosthesis, without the need for further DAIR or flap surgery or suppressive antibiotic treatment.

[Bibr bib1.bibx8] introduced the concept of a proactive flap. Among 73 patients undergoing extensive debridement after chronic TKA infection, 15 received prophylactic flap placement during initial revision arthroplasty without pre-existing defects (proactive flap) to improve soft tissue quality and blood supply. Persistent infection was observed in 58 % of patients with reactive flap placement compared to 27 % of those with proactive flap placement.

The strategy for soft tissue management in complex joint revision was outlined by [Bibr bib1.bibx10]. In their study, patients were classified based on chronology, presence of infection, and metalwork exposure. This study included all patients with soft tissue problems following joint revision, encompassing those at potential future risk and those with wound breakdown (acute or chronic).

In the group with potential soft tissue risk (Group I), patients were divided into two subgroups: those without a diagnosed infection, where an arthroplasty revision with a prophylactic flap was recommended, and those with a confirmed infection, where a two-stage revision was needed and where the flap surgery was performed in the first stage.

Patients with postoperative wound breakdown were classified as Group II or III depending on the timing. Acute or subacute cases were defined as Group II, and chronic cases were defined as Group III. In these cases, the flap was performed as a treatment for the soft tissue problems.

[Bibr bib1.bibx10] reported that patients with closed wounds had better outcomes (flap failure, persistent infection, reinfection, necessity for a second flap, or chronic wound) compared to those with open wounds, supporting the necessity for a proactive flap.

According to [Bibr bib1.bibx10], our study would be included in Group IIb – patients with acute or subacute postoperative wound breakdown with metalwork exposure. However, we divided our sample into two subgroups: those who received only soft tissue reconstruction with a flap and those who received both soft tissue reconstruction and proactive debridement, antibiotics, and implant retention (DAIR) in the same procedure. We argue that proactive DAIR during the soft tissue reconstruction, in cases of acute wound complications after total knee arthroplasty (TKA), increases favourable outcomes, supporting the notion that a persistent open wound represents a potentially infected arthroplasty, even if the diagnosis is not confirmed.

Early prosthetic joint dehiscence may indicate an acute prosthetic joint problem. Additionally, an open surgical wound could serve as an entry point for microorganisms to contact the prosthesis. This rationale underlies our hypothesis that in the context of acute soft tissue problems after TKA, a proactive approach may enhance favourable outcomes. Thus, we propose performing a “prophylactic” DAIR in conjunction with reconstruction treatment, such as a muscle flap, to address potential acute infections even when the infection diagnosis is unclear.

We could not find studies analysing the benefit of this combined approach in the same surgical setting compared to isolated treatment of soft tissue defects. In our study, the only one with this focus to our knowledge, the combination group (FLAP with DAIR) yielded better results, even with no statistical significance. The increased risk of failure in the FLAP without DAIR group compared to the isolated soft tissue reconstruction treatment was approximately with a relative risk (RR) of 2 (95 % CI: 0.75–5.33).

The risk of PJI after TKA ranges from 0.8 % to 2 % over 10 years, but it increases to approximately 3 % following aseptic prothesis exchange [Bibr bib1.bibx7]. One reason for this increase may be multiple surgeries in the same region of the body, which can affect local soft tissue vascularity. In our cohort, poorer results may partly be attributed to a greater number of knee operations before flap coverage, with a median of 2 previous surgeries before flap soft tissue reconstruction with a flap (including primary knee arthroplasty intervention). We observed a statistically significant negative relationship between multiple previous surgeries and unfavourable outcomes, with an RR of 3.75 (95 % CI: 1.02–13.8).

The literature does not define clear risk factors for gastrocnemius flap failure; however, it does identify several variables, including age, sex, obesity, medical comorbidities, diabetes, coronary artery disease, time from initial TKA to flap coverage, time from diagnosis of PJI to flap surgery, skin defect size, and number of surgeries prior to flap coverage [Bibr bib1.bibx16]. Future studies should aim to establish indications for flap coverage and develop an evidence-based reconstructive algorithm. Based on our results, we advocate for proactive DAIR (protocol FLAP with DAIR) in patients with acute soft tissue defects following TKA to address potential acute infections, even in cases where infection diagnosis is unclear.

However, we acknowledge both the strengths and limitations of our study. Among its limitations, it is a retrospective analysis with inherent biases, including sampling bias and limitations in obtaining all relevant data. Additionally, the small sample size and varying follow-up periods limit the power to detect factors predicting poorer prognosis after flap coverage. Sample size also precludes multivariate analysis to control for confounding variables. Finally, our study was conducted at a single centre, limiting external validity, although our hospital is a tertiary university hospital serving as a national reference for musculoskeletal infection units. Another limitation of the current study is that we did not collect data on postoperative knee functionality in either study group, as this was not a primary objective of the study. In future studies, it would be valuable to compare the outcomes.

More extensive studies with larger patient cohorts and longer follow-up periods are needed to generalize results to the general population.

## 5 Conclusion

In conclusion, while there was no significant statistical difference between the two groups, the promising outcomes showed that the combination treatment of DAIR with polyethylene exchange procedure and flap surgery is highly recommended in acute soft tissue defects (dehiscence or necrosis) after knee arthroplasty. However, further studies with larger sample sizes are necessary to extrapolate results to general population.

## Data Availability

Data used and analysed are available upon reasonable request.

## Appendix A

**Table A1:** Epidemiologic patient characteristics. Note that DM represents diabetes mellitus, and OAC represents oral anticoagulation.

	All patients	FLAP with DAIR	FLAP without DAIR
	N=18 (%)	N=12	N=6
Age (years)	71.4	71.8	70.7
Sex (M : F)	8:10	4:8	4:2
DM	4	3	1
OAC	6	4	2
KLICC	1.39	1.5	1.2
ASA	2.33	2.4	2.16
Prosthesis			
Primary Prosthesis	9	6	3
Revision Prosthesis	9	6	3
Fistula tract	4 (22.22)	3	1
Necrosis	13 (72.22)	9	4
Wound drainage	15 (83.33)	10	5

## Appendix B

**Table B1:** Microbiological cultures.

	All patients	FLAP with DAIR	FLAP without DAIR
	N=18	N=12	N=6
*S. epidermidis*	3	1	2
*E. coli*	2	1	1
Enterococcus	3	2	1
*K. pneumoniae*	1	0	1
*Pseudomonas aeruginosa*	1	1	0
*B. cereus*	1	1	0
Polymicrobial infection (including *Enterococcus*)	3	3	0
Culture negative	4	3	1

## Appendix C

**Table C1:** Statistical analysis.

		TKA clearance		
		No	Yes	Total
FLAP without DAIR	N	4	2	6
	%	66.7 %	33.33 %	100 %
FLAP with DAIR	N	4	8	12
	%	33.33 %	66.67 %	100 %
Total	N	8	10	18
	%	44.4 %	55.6 %	100 %

		P			95 % CI
$\chi^{2}$	1.80	P=0.180	RR	2	0.75–5.33

## References

[bib1.bibx1] Baertl S, Rupp M, Kerschbaum M, Morgenstern M, Baumann F, Pfeifer C, Worlicek M, Popp D, Amanatullah DF, Alt V (2024). The PJI-TNM classification for periprosthetic joint infections. Bone Joint Res.

[bib1.bibx2] Corten K, Struelens B, Evans B, Graham E, Bourne RB, MacDonald SJ (2013). Gastrocnemius flap reconstruction of soft tissue defects following infected total knee replacement. Bone Joint J.

[bib1.bibx3] Coughlan A, Taylor F (2020). Classifications in Brief: The McPherson Classification of Periprosthetic Infection. Clin Orthop Relat Res.

[bib1.bibx4] Galat DD, McGovern SC, Larson DR, Harrington JR, Hanssen AD, Clarke HD (2009). Surgical treatment of early wound complications following primary total knee arthroplasty. J Bone Joint Surg Am.

[bib1.bibx5] Harrison C, Alvand A, Chan J, West E, Matthews P, Taylor A, Giele H, McNally M, Ramsden A (2018). The gastrocnemius flap in the management of infected knee prostheses: experience of 115 cases over 21 years in a single centre. Orthop Proc.

[bib1.bibx6] Hotchen AJ, Wismayer MG, Robertson-Waters E, McDonnell SM, Kendrick B, Taylor A, Alvand A, McNally M (2021). The Joint-Specific BACH classification: A predictor of outcome in prosthetic joint infection. EClinicalMedicine.

[bib1.bibx7] Huotari K, Peltola M, Jämsen E (2015). The incidence of late prosthetic joint infections: A registry-based study of 112,708 primary hip and knee replacements. Acta Orthopaedica.

[bib1.bibx8] Kwiecien GJ, Lamaris G, Gharb BB, Murray T, Hendrickson MF, Zins JE, Isakow R (2016). Long-Term Outcomes of Total Knee Arthroplasty following Soft-Tissue Defect Reconstruction with Muscle and Fasciocutaneous Flaps. Plast Reconstr Surg.

[bib1.bibx9] Laing JH, Hancock K, Harrison DH (1992). The exposed total knee replacement prosthesis: a new classification and treatment algorithm. Br J Plast Surg.

[bib1.bibx10] Leckenby JI, Grobbelaar AO (2016). Strategies for Soft-Tissue Management of Complex Joint Revision Arthroplasty: A 10-Year Experience. Plast Reconstr Surg.

[bib1.bibx11] Lenguerrand E, Whitehouse MR, Beswick AD, Jones SA, Porter ML, Blom AW (2017). Revision for prosthetic joint infection following hip arthroplasty. Bone Joint Res.

[bib1.bibx12] Papaioannou K, Lallos S, Mavrogenis A, Vasiliadis E, Savvidou O, Efstathopoulos N (2010). Unilateral or bilateral V-Y fasciocutaneous flaps for the coverage of soft tissue defects following total knee arthroplasty. J Orthop Surg Res.

[bib1.bibx13] Ries MD, Bozic KJ (2006). Medial gastrocnemius flap coverage for treatment of skin necrosis after total knee arthroplasty. Clin Orthop Relat Res.

[bib1.bibx14] Sanders R, O'Neill T (1981). The gastrocnemius myocutaneous flap used as a cover for the exposed knee prosthesis. J Bone Joint Surg Br.

[bib1.bibx15] Suda AJ, Cieslik A, Grützner PA, Münzberg M, Heppert V (2014). Flaps for closure of soft tissue defects in infected revision knee arthroplasty. Int Orthop.

[bib1.bibx16] Tetreault MW, Della Valle CJ, Bohl DD, Lodha SJ, Biswas D, Wysocki RW (2016). What Factors Influence the Success of Medial Gastrocnemius Flaps in the Treatment of Infected TKAs?. Clin Orthop Relat Res.

[bib1.bibx17] Theil C, Stock ME, Gosheger G, Moellenbeck B, Schwarze J, Schmidt-Braekling T (2020). Gastrocnemius Muscle Flaps for Soft Tissue Coverage in Periprosthetic Knee Joint Infection. J Arthroplasty.

[bib1.bib1] Tornero E, Morata L, Martínez-Pastor JC, Bori G, Climent C, García-Velez DM, García-Ramiro S, Bosch J, Mensa J, Soriano A (2015). Klic: Klic-Score for Predicting Early Failure in Prosthetic Joint Infections Treated With Debridement Implant Retention and Antibiotics. Clin Microbiol Infect.

[bib1.bibx18] Warren SI, Murtaugh T, Lakra A, Reda L, Shah RP, Geller JA, Cooper HJ (2018). Treatment of Periprosthetic Knee Infection with Concurrent Rotational Muscle Flap Coverage is Associated with High Failure Rates. J Arthroplasty.

